# Cant1 Affects Cartilage Proteoglycan Properties: Aggrecan and Decorin Characterization in a Mouse Model of Desbuquois Dysplasia Type 1

**DOI:** 10.3390/biom14091064

**Published:** 2024-08-26

**Authors:** Chiara Gramegna Tota, Alessandra Leone, Asifa Khan, Antonella Forlino, Antonio Rossi, Chiara Paganini

**Affiliations:** 1 Unit of Biochemistry, Department of Molecular Medicine, University of Pavia, 27100 Pavia, Italy; chiara.gramegnatota01@universitadipavia.it (C.G.T.); alessandra.leone02@universitadipavia.it (A.L.); asifa.khan01@universitadipavia.it (A.K.); antonella.forlino@unipv.it (A.F.); 2 University School for Advanced Studies Pavia, IUSS Pavia, 27100 Pavia, Italy; 3 Centre for Inherited Diseases, Department of Research, Fondazione IRCCS Policlinico San Matteo, 27100 Pavia, Italy; chiara.paganini@unipv.it

**Keywords:** cartilage, chondrocyte, aggrecan, decorin, proteoglycan, glycosaminoglycan, skeletal disorders

## Abstract

Desbuquois dysplasia type 1 (DBQD1) is a recessive chondrodysplasia caused by mutations in the *CANT1* gene, encoding for the Golgi Calcium-Activated Nucleotidase 1 (CANT1). The enzyme hydrolyzes UDP, the by-product of glycosyltransferase reactions, but it might play other roles in different cell types. Using a *Cant1* knock-out mouse, we demonstrated that CANT1 is crucial for glycosaminoglycan (GAG) synthesis; however, its impact on the biochemical properties of cartilage proteoglycans remains unknown. Thus, in this work, we characterized decorin and aggrecan from primary chondrocyte cultures and cartilage biopsies of mutant mice at post-natal day 4 by Western blots and further investigated their distribution in the cartilage extracellular matrix (ECM) by immunohistochemistry. We demonstrated that the GAG synthesis defect caused by CANT1 impairment led to the synthesis and secretion of proteoglycans with shorter GAG chains compared with wild-type animals. However, this alteration did not result in the synthesis and secretion of decorin and aggrecan in the unglycanated form. Interestingly, the defect was not cartilage-specific since also skin decorin showed a reduced hydrodynamic size. Finally, immunohistochemical studies in epiphyseal sections of mutant mice demonstrated that the proteoglycan structural defect moderately affected decorin distribution in the ECM.

## 1. Introduction

The extracellular matrix (ECM) of cartilage is characterized by two major structural components: collagens and proteoglycans (PGs). Collagen fibrils are heteropolymers that include different collagen types: collagen type II is the predominant component, but collagens type IX and XI are also present [1]. Similarly, the PG fraction contains the cartilage-specific PG aggrecan and, in addition, the small leucine-rich proteoglycans (SLRPs) decorin, biglycan, and fibromodulin [2]. PGs are macromolecules composed of a core protein to which glycosaminoglycans (GAGs), linear polysaccharide chains with variable length and composition, are covalently attached [3]. To date, 43 distinct PG encoding genes and many alternatively spliced variants have been characterized [4].

The biosynthesis of PGs, as well as most ECM components, occurs through the endoplasmic reticulum (ER) and the Golgi apparatus and involves the interaction between numerous molecular players along the secretory pathway. Several steps of ECM protein processing and maturation are met by the Golgi complex, which acts as a hub for post-translational modifications and sorting of cargo within the secretory pathway [5,6]. The importance of Golgi organization and function for PG glycosylation and sulfation is highlighted by the numerous disorders, primarily impacting cartilage and bone, caused by defects in PG post-translational modifications. They are grouped in (i) disorders linked to proteins involved in the synthesis of the tetrasaccharide linker region occurring in the cis-medial Golgi, (ii) disorders linked to proteins involved in the elongation of GAG chains occurring in the medial-trans Golgi, and (iii) disorders linked to proteins involved in sulfation of GAG chains occurring mainly in the trans Golgi [7].

Desbuquois dysplasia type 1 (DBQD1) (MIM 251450) belongs to the disorders linked to GAG chain synthesis and elongation. Patients are characterized by severe pre- and post-natal growth retardation with short extremities, joint laxity, and progressive scoliosis. The main radiologic features include short long bones, a “Swedish key” appearance of the proximal femur, and specific anomalies at the extremities, including advanced carpal and tarsal ossification with extra ossification centers and delta phalanx [8]. Additionally, a variant known as the “Kim variant” has been identified, characterized by almost normal hands but significant radiographic changes, including short metacarpals, elongated phalanges, and advanced carpal bone age [9]. DBQD1 is caused by mutations in the *Calcium Activated Nucleotidase 1* (*CANT1*) gene encoding for an ER and Golgi nucleoside diphosphatase that preferentially hydrolyzes UDP to UMP and phosphate [10,11,12]. Due to its substrate preference, its localization, and the patients’ clinical features, it has been suggested that CANT1 plays a pivotal role in PG synthesis through the hydrolysis of UDP, a by-product of glycosyltransferase reactions. UDP removal is essential for glycosyltransferase activity, preventing their inhibition and allowing for the exchange of UMP with cytosolic UDP sugars through an antiporter exchanger. Interestingly, fibroblasts from DBQD1 patients showed reduced PG synthesis, in particular, after incubation with β-D-xyloside, a compound that enhances GAG synthesis [13].

To define the molecular basis of DBQD1, a *Cant1* knock-out mouse (*Cant1^−/−^*) recapitulating the human phenotype was generated. Using this model, the crucial role of CANT1 in cartilage PG synthesis and endochondral ossification was demonstrated [14]. Specifically, GAG synthesis was decreased in chondrocytes from *Cant1^−/−^* mice, and overall PG secretion was delayed. Interestingly, knock-out chondrocytes had dilated ER cisternae, suggesting delayed protein secretion and ER stress; however, no canonical unfolded protein response (UPR) was detected. The observed PG defects caused deregulated chondrocyte proliferation and apoptosis in the growth plate, resulting in reduced skeletal growth [14]. These findings were further confirmed in the *Cant1* knock-out mouse generated using CRISPR/Cas9 mediated genome editing [15].

In conclusion, CANT1 plays a role in GAG chain synthesis and elongation, but its impact on the biochemical properties of cartilage PGs remains unknown. To fill the gap, in this work, we have characterized decorin and aggrecan from primary chondrocyte cultures and cartilage biopsies of *Cant1* knock-out mice and investigated their distribution in the cartilage ECM. We have demonstrated that both PGs are secreted in the ECM in the glycanated form either in vitro or ex vivo; however, in mutant animals, a shortened GAG chain is bound to the decorin core. Moreover, the defect is not cartilage-specific since skin decorin also shows the same alteration.

## 2. Materials and Methods

### 2.1. Animal Model and Care

In this study, wild-type and homozygous *Cant1* knock-out mice with a C57Bl/6Jx129/SV background were used [14]. To discriminate homozygous mutants from heterozygous and wild-type animals, mice were genotyped by polymerase chain reaction (PCR) using genomic DNA extracted from mouse tail clips.

The care and use of animals complied with relevant animal welfare institutional guidelines and protocols and were approved by the Animal Care and Use Committee of the University of Pavia and the Ministry of Health (Licence n. 486/2019-PR).

### 2.2. Chondrocyte Cultures

Chondrocytes were isolated from the rib cartilages of post-natal day (P)4 mice, as previously described [16]. Mice were sacrificed, and their thoracic cage was harvested and digested with 2 mg/mL collagenase type II (Invitrogen, ThermoFisher, Waltham, MA, USA) in Dulbecco’s modified Eagle’s medium (DMEM, Merck, Milan, Italy) at 37 °C for 60 min, to remove intercostal muscles. Then, cartilages were dissected from each rib using the dissecting microscope and digested with 2 mg/mL collagenase type II in DMEM with 10% fetal bovine serum (FBS, Euroclone, Milan, Italy) at 37 °C overnight. Released chondrocytes were cultured in DMEM with 10% FBS and antibiotics at 37 °C in a humidified atmosphere containing 5% CO_2_.

### 2.3. Protein Extraction from Chondrocytes

For decorin and aggrecan Western blot analysis, chondrocytes were plated in 60 mm diameter petri dishes at a cell density of 1.5 × 10^6^ cells/petri in DMEM with 10% FBS and incubated at 37 °C in 5% CO_2_. Two days after seeding, cells were incubated in DMEM without FBS at 37 °C in 5% CO_2_ for 24 h. The medium was then harvested and ultrafiltered with Amicon Ultra 4 mL Centrifugal Filter unit (10 kDa cut-off, Merck, Milan, Italy) at 4200× *g* for 10 min. For buffer exchange, the Centrifugal Filter unit was washed with 0.1 M ammonium acetate, pH 7.35, centrifuged at 4200× *g* for 10 min, and the retentate was lyophilized. The cell layer was scraped in PBS, centrifuged at 12,000× *g* for 5 min at 4 °C, and the pellet was resuspended in RIPA lysis buffer (150 mM NaCl, 1% IGEPAL CA-630, 0.5% sodium deoxycholate, 0.1% sodium dodecylsulphate (SDS), 50 mM Tris-HCl, pH 8.0) and solubilized with 3 cycles of freezing and thawing. Lysates were clarified by centrifugation at 12,000× *g* for 5 min at 4 °C, and the protein content was measured by the BCA Protein Assay (ThermoFisher Scientific, Waltham, MA, USA) using bovine serum albumin (BSA, ThermoFisher Scientific, Waltham, MA, USA) as standard. For buffer exchange in 0.1 M ammonium acetate, pH 7.35, samples were ultrafiltered with Amicon Ultra 0.5 mL Centrifugal Filter units (10 kDa cut-off, Merck, Milan, Italy) at 9000× *g* for 10 min. The retentates were lyophilized without chondroitinase ABC digestion to analyze the native form of intracellular and secreted decorin. For decorin analysis, lyophilized samples were resuspended in Laemmli buffer (62.5 mM Tris-HCl, 2% SDS, 10% glycerol, 0.01% bromophenol blue) containing 5% β-mercaptoethanol and denatured at 90 °C for 10 min. In preparation for aggrecan immunoblots, it was necessary to unmask the epitope on the aggrecan core protein by digestion with chondrotinase ABC to remove GAGs [17]. For this purpose, lyophilized samples were dissolved in 0.1 M ammonium acetate, pH 7.35, and divided into two aliquots; one aliquot was digested with 40 mU of chondroitinase ABC (Merck, Milan, Italy) in 0.1 M ammonium acetate pH 7.35 to unmask the epitope on the aggrecan core protein, while the other aliquot was left undigested. All samples were incubated overnight at 37 °C, then lyophilized, denatured in Laemmli buffer containing 5% β-mercaptoethanol, and analyzed by Western blot.

### 2.4. Proteoglycan Extraction from Cartilage Femoral Head and Skin

For decorin and aggrecan Western blot analysis, proteins were extracted from femoral heads and skin biopsies of P4 mice; for each sample, femoral heads from 4 mice were pooled. Samples were homogenized in urea buffer (8 M urea, 50 mM Tris-HCl, pH 7.4) with the Ultra-Turrax T-8 (IKA, Staufen, Germany), and proteins were extracted by stirring overnight at 4 °C. The homogenate was clarified by centrifugation at 12,000 rpm for 15 min at 4 °C, and the pellet was extracted again in urea buffer by stirring overnight at 4 °C. The second extract was clarified by centrifugation and pooled to the first extract, while the pellet was used for collagen extraction. Samples were precipitated with 9 volumes of 96% ethanol overnight at 4 °C, centrifuged at 12,000 rpm for 20 min at 4 °C, and the pellet was washed with 70% ethanol. Then, samples were resuspended in 0.1 M ammonium acetate pH 7.35 and sonicated to completely dissolve the pellets. The protein content was measured by the BCA Protein Assay, using BSA as standard. To unmask the epitope on the aggrecan core protein and to study the decorin core protein by digestion with chondroitinase ABC, samples were split into two aliquots: one aliquot was digested with 40 mU of chondroitinase ABC (Merck, Milan, Italy) in 0.1 M ammonium acetate, pH 7.35, to remove GAGs and unmask the core protein [17], while the latter aliquot was left undigested. All samples were incubated overnight at 37 °C, then lyophilized, denatured in Laemmli buffer containing 5% β-mercaptoethanol, and analyzed by Western blot.

### 2.5. Purification of the Core Protein Standard from Cartilage Proteoglycans

A standard of PG core proteins from mouse cartilage was used as a positive control for aggrecan and decorin Western blots. The cartilage femoral heads from 10 wild-type P4 mice were homogenized in 3 mL of 8 M urea, 50 mM sodium acetate buffer, pH 6.0, 0.15 M NaCl, 0.5% Triton X-100 supplemented with proteinase inhibitors, and proteins were extracted by stirring overnight at 4 °C. The extract was clarified by centrifugation at 12,000 rpm, and the supernatant was loaded on a 1 mL DEAE Sephacel™ (Cytiva Italy, Milan, Italy) column. After column washing with 50 mM sodium acetate buffer, pH 6.0, 8 M urea, 0.15 M NaCl, 0.5% Triton X-100, and proteinase inhibitors, PGs were eluted with 1 M NaCl in the same buffer and recovered by precipitation with 9 volumes of 96% ethanol overnight at 4 °C. The sample was centrifuged at 12,000 rpm with a Sorvall SS-34 rotor (Thermo Fisher Scientific, Waltham, MA, USA) at 4 °C for 1 h. The supernatant was discharged, while the pellet was washed with 70% ethanol and then dissolved in 0.1 M ammonium acetate buffer, pH 7.35, containing 80 mU chondroitinase ABC (Merck, Milan, Italy) to digest chondroitin/dermatan sulfate GAGs. Digestion was performed at 37 °C overnight. Then, samples were lyophilized, dissolved in Laemmli buffer, and denatured at 90 °C for 10 min.

### 2.6. Western Blot Analysis

Samples from PG extraction were analyzed by 10% SDS-PAGE and 4–15% gradient SDS-PAGE (Bio-Rad, Milan, Italy) for decorin and aggrecan analysis, respectively. Proteins were transferred to the polyvinylidene difluoride (PVDF) membrane (Amersham Biosciences, Amersham, UK). Membranes were blocked with 5% BSA (Merck, Milan, Italy), 0.05% Tween-20 (Merck, Milan, Italy) in Tris-buffered saline (TBS) and incubated with primary antibodies against decorin (1:500, goat polyclonal antibody cat. n. AF1060, R&D System, Minneapolis, MN, USA), aggrecan (1:500, rabbit monoclonal antibody cat. n. AB1031, Merck, Milan, Italy), β-actin (1:5000 mouse monoclonal antibody cat. n. MAB1501, Merck, Milan, Italy), and the appropriate peroxidase-conjugated secondary antibody (1:2000 rabbit anti-goat antibody cat. n. sc-2777, Santa Cruz Biotechnology, Dallas, TX, USA; 1:2000 goat anti-rabbit antibody cat. n. 7074 and 1:2000 horse anti-mouse antibody cat. n. 7076, Cell Signaling, Danvers, MA, USA). Membranes were visualized by chemiluminescence with Westar C 2.0 (Cyanagen, Bologna, Italy), and images were acquired by ImageQuant LAS 4000 (GE Healthcare Bio-Sciences, Piscataway, NJ, USA). For aggrecan cell layer samples, the aggrecan band was normalized to β-actin. For normalization of aggrecan and decorin medium samples, before the blocking step, the membrane was stained with the Swift Membrane Stain™ (G-Biosciences, St. Louis, MO, USA) according to the manufacturer’s instructions and scanned with ImageQuant LAS 4000 (GE Healthcare Bio-Sciences, Piscataway, NJ, USA).

### 2.7. Analysis of Collagen from Femoral Head and Skin

Collagen was purified by digestion of the cartilage and skin pellets left after PG extraction with 100 μg/mL pepsin (Merck, Milan, Italy) in 0.5 M acetic acid, pH 2.0, overnight at 4 °C, followed by precipitation with an equal volume of 4 M NaCl in 1 M acetic acid overnight at 4 °C. Samples were then centrifuged at 12,000 rpm for 20 min at 4 °C, the supernatant was discarded, and the pellet was washed with 70% ethanol, dissolved in 0.5 M acetic acid, and lyophilized. The lyophilizate was weighed, dissolved in Laemmli buffer to a concentration of 2 μg/μL, and denatured at 90 °C for 10 min.

Collagen was analyzed by 6% SDS-PAGE in non-reducing conditions, and gels were stained with Coomassie Brilliant Blue R-250 (Bio-Rad, Milan, Italy). Images were acquired using the Chemidoc XRS apparatus (Bio-Rad, Milan, Italy).

### 2.8. Immunohistochemical Analysis

After sacrifice, the hind limbs of P4 mice were fixed in 10% formalin and then decalcified in MicroDec, EDTA-based (Diapath S.p.A., Martinengo, Italy) overnight. Samples were dehydrated with an increasing series of ethanol and embedded in paraffin. Five μm sections were cut parallel to the long axis of the tibia using an RM2265 microtome (Leica Microsystems, Milan, Italy). For immunohistochemical analysis, sections were deparaffinized in xylene and rehydrated in decreasing concentrations of ethanol; then, sections were washed in PBS (Merck, Milan, Italy) for 10 min. For aggrecan and decorin analysis, sections were incubated in 0.1 M ammonium acetate, pH 7.35, for 2 min at room temperature and then digested with 0.5 U/mL chondroitinase ABC (Merck, Milan, Italy) in 0.1 M ammonium acetate buffer, pH 7.35, for 1 h at 37 °C to unmask the epitope on the core protein. For collagen type II analysis, sections were digested with 250 µg/mL pepsin (Merck, Milan, Italy) in 0.2 N HCl for 15 min at 37 °C. Sections were then incubated with hyaluronidase buffer (150 mM sodium chloride, 5 mM EDTA, 2.5 mM benzamidine hydrochloride and 10 µg/mL soybean tripsin inhibitor in 50 mM sodium acetate, pH 5.5) for 2 min at room temperature and digested with 2 mg/mL bovine testes hyaluronidase (Merck, Milan, Italy) in hyaluronidase buffer for 45 min at 37 °C. After the unmasking step, sections were washed twice with PBS and incubated with 3% hydrogen peroxide for 10 min at room temperature to block endogenous peroxidase activity. Then, slides were washed twice with PBS and blocked in 5% BSA in PBS for 1 h at room temperature. After two washings with PBS, sections were incubated overnight at 4 °C with the primary antibody in 1% BSA in PBS (1:25 rabbit anti-aggrecan antibody cat. n. AB1031, Merck, Milan, Italy; 1:50 goat anti-decorin antibody cat. n. AF1060, R&D Systems, Minneapolis, MN, USA; 1:500 mouse anti-collagen type II antibody cat. n. CP18, Merck, Milan, Italy). Then, sections were washed twice with PBS-T (0.05% Tween 20 in PBS) and incubated with the appropriate peroxidase-conjugated secondary antibody diluted in 1% BSA in PBS (1:500 anti-rabbit antibody cat. n. 31460, Invitrogen, ThermoFisher, Waltham, MA, USA; 1:500 anti-goat antibody cat. n. 31402, Invitrogen, ThermoFisher, Waltham, MA, USA; 1:500 anti-mouse antibody cat. n. 31430, Invitrogen, ThermoFisher, Waltham, MA, USA) for 1 h at room temperature. After two washings with PBS-T and PBS, antigen detection was performed using the DAB Substrate Detection Kit (ThermoFisher, Waltham, MA, USA) according to the manufacturer’s instructions. Then, sections were washed with PBS, dehydrated, and mounted. Images were acquired at Centro Grandi Strumenti of the University of Pavia using a Widefield Leica microscope (DM6B) driven by LAS X software version 3.4.2 (Leica Microsystems, Milan, Italy), equipped with a DFC7000 T camera (Leica Microsystems, Milan, Italy) and 4×, 20× and 40× objectives.

## 3. Results

### 3.1. Characterization of Decorin in Cant1 Knock-Out Chondrocytes and Tissues

**Figure 1:**
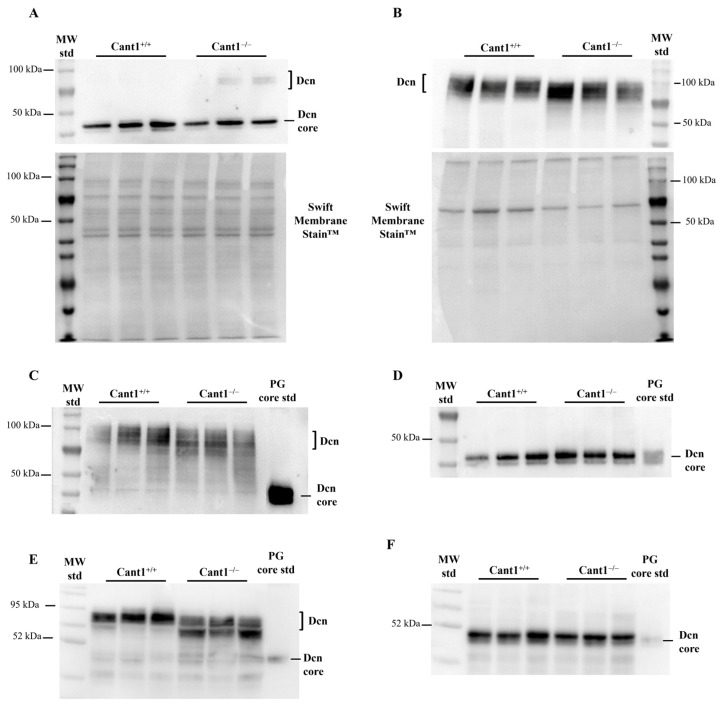
Western blot analysis of decorin in chondrocyte cultures and cartilage and skin. (**A**) Decorin Western blot of cell lysates and (**B**) culture medium from *Cant1* knock-out (Cant1^−/−^) and wild-type (Cant1^+/+^) chondrocytes (n = 3). Membrane staining with Swift Membrane Stain™ was used as loading control. (**C**) Decorin Western blot of femoral head cartilage extracts from *Cant1* knock-out (Cant1^−/−^) and wild-type (Cant1^+/+^) mice (n = 3). For each sample, femoral heads from 4 mice were pooled. (**D**) Decorin Western blot of the same samples shown in (**C**) after digestion with chondroitinase ABC to release the decorin core protein (n = 3). (**E**) Decorin Western blot of skin extracts from *Cant1* knock-out (Cant1^−/−^) and wild-type (Cant1^+/+^) mice (n = 3). (**F**) Decorin Western blot of the same samples shown in (**E**) after digestion with chondroitinase ABC to release the decorin core protein (n = 3). In panel A, the same protein amount from each sample (20 µg) was loaded, while in panel B, the same volume from each sample (20 µL) was loaded. In panels (**C**–**F**), the same protein amount was loaded from each sample (10 µg). Dcn: decorin; PG core std: standard of cartilage PG core proteins purified as described in Materials and Methods. Panels (**A**,**B**) and panels (**C**–**F**) are representative experiments of three and two technical replicates, respectively.

In previous works, we demonstrated that Cant1 was important for chondroitin/dermatan sulfate synthesis [13,14]. In order to study how GAG synthesis impairment impacted decorin synthesis and secretion in mutant mice, we studied this PG in primary cultures of rib chondrocytes from P4 mice. Western blot on cell lysates and medium were performed using an antibody raised against the decorin core that allows for the detection of both the glycanated and unglycanated forms. In cell lysates, a band at about 40 kDa, corresponding to the decorin core, was present both in wild-type and mutant cells, indicating that this PG was present inside the cells, mainly in the unglycanated form (Figure 1A), while only a broad band (MW 70–100 kDa) corresponding to the glycanated form was present in the medium, indicating that the GAG synthesis defect in mutant chondrocytes did not result in the secretion of the free core protein and only the glycanated PG was secreted in the ECM (Figure 1B). Occasionally, a faint, not quantifiable band of glycanated decorin was observed in mutant cell lysates. Interestingly, the electrophoretic migration of extracellular decorin from mutant cells was faster, with a greater proportion of lower molecular weight components compared with wild-type cells, while the decorin core protein was normal in size (Figure 1A). This result demonstrated that in mutant cells, the length and, thus, the hydrodynamic size of GAG chains were reduced compared with wild-types.

To confirm the in vitro results, femoral head cartilage from mutant and wild-type P4 animals was homogenized in 8 M urea, 50 mM Tris-HCl, pH 7.4, and total proteins recovered by precipitation with nine volumes of ethanol. Western blot analysis demonstrated that only glycanated decorin was present in the cartilages of both mutant and wild-type P4 mice (Figure 1C). As observed in vitro, glycanated decorin in mutant samples showed a broad band with a lower apparent molecular weight, suggesting shortened GAG chains, while the decorin core protein was normal in size after digestion with chondroitinase ABC (Figure 1D). In order to check whether the decorin glycanation defect was cartilage-specific, decorin purified from the skin of P4 mice was studied by Western blot. Also, in this tissue, the results mirrored cartilage data: glycanated decorin showed a lower apparent molecular weight in mutants compared with wild-type animals (Figure 1E), while the decorin core was normal (Figure 1F), suggesting that the glycanation defect was not restricted to cartilage.

### 3.2. Characterization of Aggrecan in Cant1 Knock-Out Chondrocytes and Cartilage

**Figure 2:**
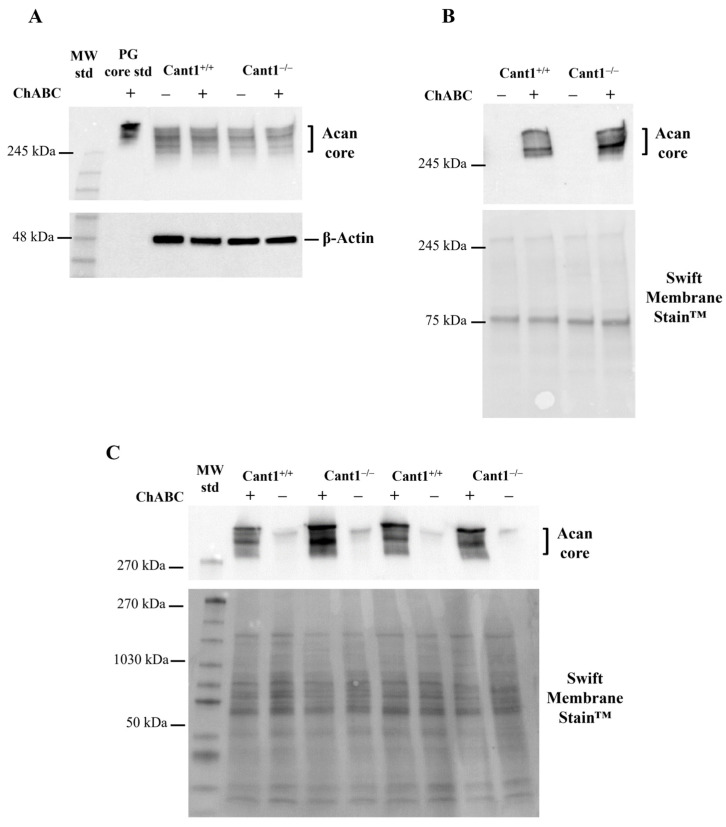
Western blot analysis of aggrecan in chondrocyte cultures and in cartilage of P4 *Cant1* knock-out (Cant1^−/−^) and wild-type (Cant1^+/+^) mice. (**A**) Aggrecan Western blot of chondrocyte lysates not digested (−) or digested (+) with chondroitinase ABC to release the core protein; β-actin was used as loading control (n = 3). (**B**) Aggrecan Western blot of chondrocyte culture medium not digested (−) or digested (+) with chondroitinase ABC to release the core protein (n = 3). Membrane staining with Swift Membrane Stain™ was used as loading control. (**C**) Aggrecan Western blot of femoral head cartilage extracts from *Cant1* knock-out (Cant1^−/−^) and wild-type (Cant1^+/+^) mice not digested (−) or digested (+) with chondroitinase ABC to release the core protein (n = 3). Membrane staining with Swift Membrane Stain™ was used as loading control. For each sample, femoral heads from 4 mice were pooled. In panels (**A**, **C**), the same protein amount was loaded from each sample (20 µg for chondrocyte lysates and 10 µg for femoral head cartilage samples), while in panel (**B**), the same volume from each sample (20 µL) was loaded. Acan: aggrecan; PG core std: standard of cartilage PG core proteins purified as described in Materials and Methods; ChABC: chondroitinase ABC. Panels (**A**,**B**) and panel (**C**) are representative experiments of three and two technical replicates, respectively.

Aggrecan is a cartilage-specific PG that plays a variety of important functions in articular and growth plate cartilage. Thus, we studied its biochemical properties in cartilage and chondrocyte cultures when Cant1 is impaired. As it was performed for decorin studies, aggrecan synthesized by rib chondrocytes from mutant and wild-type mice was analyzed. It was not possible to study the glycanated aggrecan form by immunoblots because the aggrecan epitope residing in the core protein was masked by GAGs; for this reason, only the unglycanated form or the core, unmasked by chondroitinase ABC digestion, was detectable. Aggrecan in medium and cell lysates from mutant and wild-type primary chondrocytes was analyzed by Western blot before and after digestion with chondroitinase ABC. The core protein was detected in cell lysates, even in samples that were not digested with chondroitinase ABC (Figure 2A). Moreover, equal loading of cell lysates undigested or digested with chondroitinase ABC showed an aggrecan core protein band with the same intensity and electrophoretic mobility in both mutant and wild-type cells, indicating that aggrecan was present in its non-glycanated form inside the cells. In the medium of both mutant and wild-type cells, a broad band with an apparent size >250 kDa was observed after digestion with chondroitinase ABC corresponding to the aggrecan core protein (Figure 2B). No signal was detected in undigested samples, indicating that the aggrecan core protein was not secreted in the culture medium of the mutant, as well as the wild-type cells.

Aggrecan studies by Western blots were also performed in the cartilage femoral heads of P4 animals. Cartilage samples were homogenized in 8 M urea, 50 mM Tris-HCl, pH 7.4; total proteins, recovered by nine volumes of ethanol precipitation, were divided into two aliquots that were digested or not digested with chondroitinase ABC followed by Western blot with the aggrecan antibody. In mutant and wild-type tissues, the aggrecan core was detected in very high abundance only after digestion with chondroitinase ABC, indicating that, as already observed in cell cultures, the glycanated form was present in the ECM (Figure 2C). In both mutant and wild-type cartilages, a similar faint signal pointing to a trace amount of aggrecan core protein was observed in the not-digested samples; this form might come from ECM degradation and/or intracellularly since P4 epiphyseal cartilage has high cellularity compared with adult articular cartilage.

Interestingly, staining of the proximal epiphysis with toluidine blue, a cationic dye for GAGs, confirmed Western blot results. Indeed, no metachromatic differences were observed between mutant and wild-type epiphysis, demonstrating that PGs were present in the glycanated form in the cartilage ECM from both genotypes (Supplementary Figure S1).

### 3.3. Collagen Analysis in Cartilage and Skin

**Figure 3:**
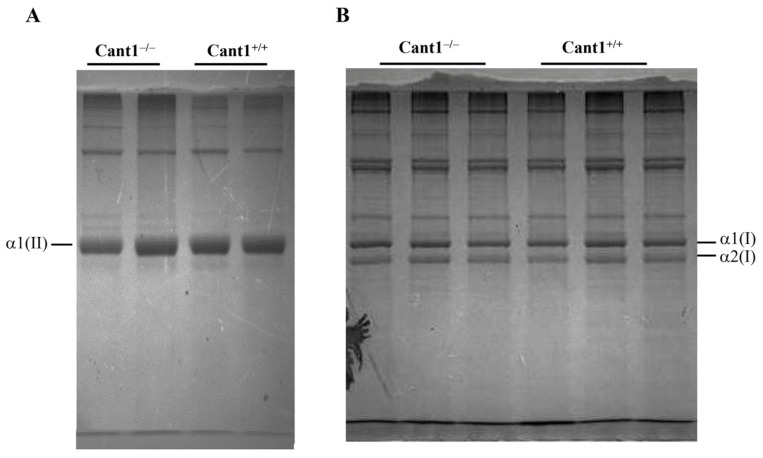
Electrophoresis of pepsin-purified collagens from cartilage and skin. (**A**) 6% SDS-PAGE of collagens purified from femoral head cartilage of *Cant1* knock-out (Cant1^−/−^) and wild-type (Cant1^+/+^) P4 mice (n = 2). For each sample, femoral heads from 4 mice were pooled. (**B**) 6% SDS-PAGE of collagens purified from skin of *Cant1^−/−^* and *Cant1^+/+^* P4 mice (n = 3). Gels are representative experiments of three technical replicates.

Besides PGs, collagen is the other predominant component of the cartilage ECM. Even if collagen glycosylation occurs in the ER, we checked whether Cant1 might affect collagen post-translational modifications in cartilage and skin from mutant and wild-type P4 animals. After femoral head cartilage or skin extraction in 8 M urea for PG purification, the insoluble pellets were digested with pepsin. Pepsin-solubilized collagens were then precipitated with 2 M NaCl and analyzed by 6% SDS-PAGE, followed by Coomassie Brilliant blue staining. Electrophoretic analysis of cartilage samples showed the α1(II) band in mutant and wild-type samples, indicating normal expression of collagen type II (Figure 3A). The electrophoretic mobility of the collagen band in mutant animals was normal, suggesting that no gross collagen glycosylation defects were present due to Cant1 impairment. These results were further confirmed by electrophoretic analysis of skin collagen from mutant and wild-type mice; also, in these samples, normal migrating bands corresponding to the α1(I) and α2(I) chains of collagen type I were observed (Figure 3B).

### 3.4. Immunohistochemical Localization of Decorin, Aggrecan, and Collagen Type II in the Tibia Epiphysis

**Figure 4:**
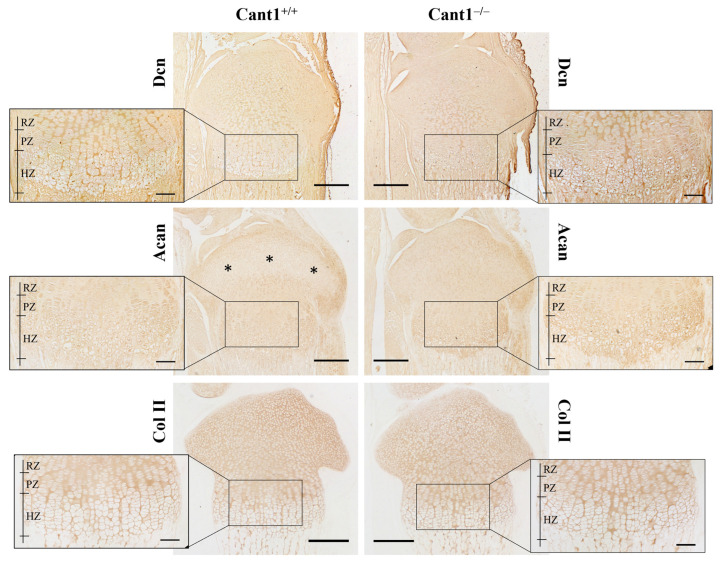
Immunohistochemistry of the proximal tibia epiphysis of P4 *Cant1* knock-out (Cant1^−/−^) and wild-type (Cant1^+/+^) mice. Sections were immunostained for decorin (Dcn), aggrecan (Acan), and collagen type II (Col II). Asterisks in WT Acan panel point to the area, giving rise to the secondary ossification center. Representative images from the analysis of 3 mice for each genotype. RZ = resting zone; PZ = proliferative zone; HZ = hypertrophic zone. Scale bar in 4× images = 300 µm; scale bar in 20× images = 100 µm.

**Figure 5:**
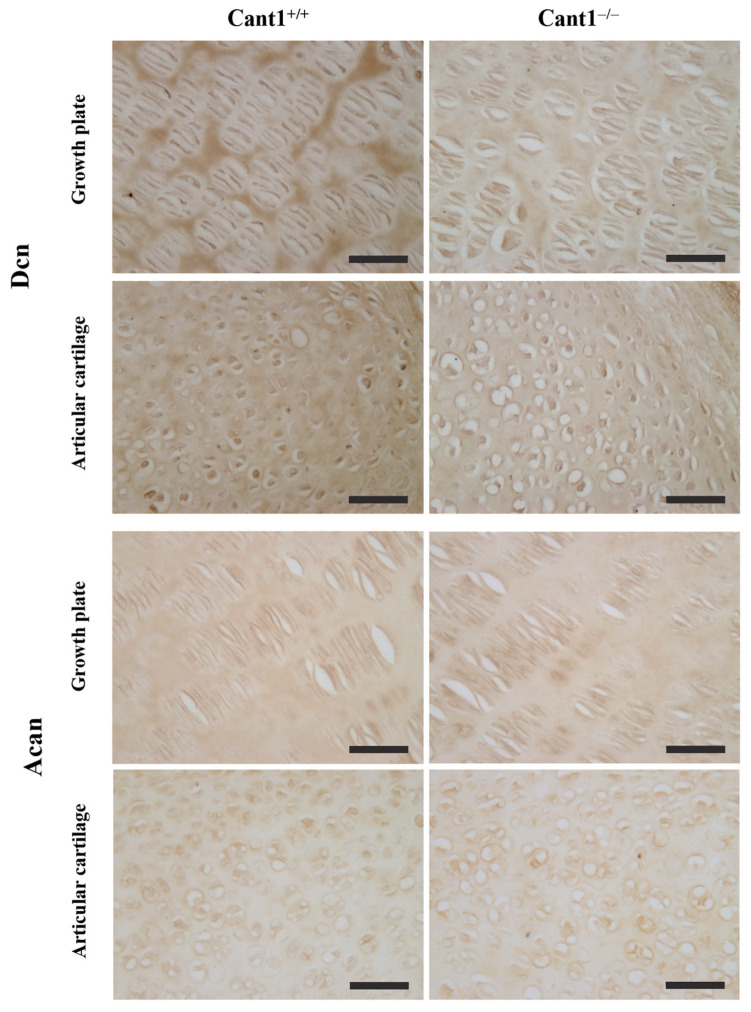
High magnification (40×) of immunohistochemistry sections shown in Figure 4 from the proximal tibia epiphysis of *Cant1* knock-out (Cant1^−/−^) and wild-type (Cant1^+/+^) P4 mice. Sections were immunostained for decorin (Dcn) or aggrecan (Acan). Representative images of the proliferative zone of the growth plate and the articular cartilage from the analysis of 3 mice for each genotype. Scale bar: 5 µm.

The tibia epiphyseal growth plate of *Cant1* knock-out mice at P7, 14, and 21 has already been described [14]. While the overall architecture is maintained with clearly defined resting, proliferative, and hypertrophic zones, the area of the proliferative and hypertrophic zones at P7 is reduced, as well as the number of cells per column. Since in this study, we analyzed PGs and collagen from the cartilage of mutant and wild-type P4 mice, we investigated the distribution of these proteins in the tibia epiphysis at the same age point using specific antibodies (Figure 4). In wild-type and mutant animals, an even distribution of decorin was observed in the resting and articular zones. At high magnification in the proliferative zone, a more abundant decorin staining was observed between the chondrocyte columns in wild-type compared with mutant animals, suggesting an altered decorin distribution among the interterritorial and pericellular matrix of the growth plate that was not evident in articular cartilage (Figure 5).

For aggrecan immunostaining, tibia slides were digested with chondroitinase ABC to unmask the aggrecan core protein before incubation with the specific antibody. In mutant animals, aggrecan showed an even distribution in the whole epiphysis with a positive signal both in the interterritorial and pericellular matrix (Figure 4 and Figure 5). In wild-type animals, a reduced aggrecan signal was observed in the epiphyseal area, which later gave rise to the secondary ossification center (Figure 4).

For collagen type II immunohistochemistry, tissue sections were unmasked by digestion with pepsin, followed by incubation with a collagen type II antibody. In both mutant and wild-type slides, no relevant differences were observed, with an even distribution of collagen type II in the ECM of the epiphysis (Figure 4).

## 4. Discussion

According to the latest Nosology of genetic skeletal disorders revised in 2023, which includes more than 750 different entities, DBQD1 belongs to Group 5, “Dysplasias with multiple joint dislocations” [18]. This group contains several disorders of GAG synthesis, and it is phenotypically related to Group 4, which includes sulfation disorders of GAGs. Based on the patients’ clinical phenotype similarities and substrate preferences, a role for CANT1 in GAG synthesis was inferred [11] and subsequently demonstrated [13,14]. However, the exact function(s) of CANT1 and its specific role in the pathogenesis of DBQD1 is far from complete.

CANT1 is a nucleotidase that has been localized in the ER and Golgi; however, indirect evidence suggests that it might work in the Golgi. In fact, Trombetta et al. identified in the ER a soluble UDP nucleotidase (ENTPD5) that was clearly distinct from the nucleoside diphosphatase activity of the Golgi enzyme [19]. *Entpd5* knock-out mice are viable but develop progressive hepatopathy, hepatocellular tumors, and spermatogenic arrest [20]; however, no skeletal alteration has been reported. Thus, CANT1 might be the ENTPD5 homolog that works mainly in the Golgi and is involved in protein glycosylation.

It has been demonstrated that CANT1 is involved in tumor growth: it is overexpressed in prostate cancer, lung squamous cell carcinoma, lung adenocarcinoma, and hepatocellular carcinoma [21,22,23]. Further studies in neuroblastoma cells have demonstrated that CANT1 is a target gene of the DREAM protein, a Ca^2+^-dependent transcriptional repressor, and its activity is essential to the correct ER protein folding and degradation processes [24]. It has been reported that calcium induced the dimerization of recombinant CANT1, and the dimeric form exhibited higher nucleotidase activity compared with the monomer, suggesting that at the high Ca^2+^ concentrations present in the Golgi, the enzyme may always be highly active [25,26]. These changes linked to the dimerization status may regulate enzyme activity and its function in glycosylation reactions. Thus, CANT1 might play multiple roles in different cell types due also to the pleiotropic functions of the Golgi, which is not only the core organelle for post-translational modifications and the secretory pathway but also has a major role in signaling, autophagy, and apoptosis [5,27].

Recently, significant advances in the development of therapeutic approaches for skeletal disorders have been achieved with the aim of alleviating, preventing, or modifying the disease progression [28,29,30,31,32,33]. These progresses have been made thanks to deep phenotyping of in vitro and/or in vivo models that have allowed for the identification of new molecules or already existing ones through a drug repositioning approach. For this reason, there is an urgent need to elucidate the exact function(s) of CANT1 in skeletal development, growth, and homeostasis and to explore the molecular mechanisms causing DBQD1 in order to identify new therapeutic targets for pharmacological treatments. We have recently generated a *Cant1* knock-in and knock-out mouse that we have validated as in vivo models of DBQD1 [14]. Focusing on the *Cant1* knock-out that was used in this work, a skeletal phenotype with reduced growth, moderate thoracic kyphosis, and abnormalities in bone extremities has been demonstrated. Overall, GAG synthesis was decreased in knock-out chondrocytes, and their hydrodynamic size was reduced. The defect was boosted when cells were incubated with β-D-xyloside, an enhancer of GAG synthesis.

In this work, to further characterize how GAG synthesis impairment affects PG synthesis and distribution in the ECM, we have characterized aggrecan and decorin in the epiphyseal cartilage of *Cant1* knock-out mice.

Aggrecan consists of a ~250 kDa core protein decorated with more than 100 chondroitin sulfate GAG chains. In cartilage, more than 90% of chondroitin sulfate GAGs are present in aggrecan molecules, providing the ECM with its negatively charged environment [34]. Decorin bears a core protein composed of leucine-rich repeat structures that interact with collagen matrix glycoproteins and plasma membrane components [3]. It is linked to one chondroitin/dermatan sulfate chain, accounting for further interactions. Moreover, on a molar basis, the decorin and aggrecan concentrations are similar [35].

Through SDS-PAGE followed by immunoblotting with a specific decorin antibody, we have demonstrated that decorin secreted by mutant chondrocytes or present in the cartilage ECM bears, on average, a reduced hydrodynamic size compared with wild-types. The difference was due to the reduced length of the GAG chain since the core protein released after digestion with chondroitinase ABC was normal. Moreover, in both mutant and wild-type chondrocytes, the analysis of the cell lysate and medium fraction showed that glycanated decorin was secreted, while in cell lysates of mutant and wild-type cells, the unglycanated form was present. It is worth noting that in other disorders affecting PG biosynthesis, it has been demonstrated that the defect also caused the secretion of the decorin core protein without the oligosaccharide chain. In fibroblasts from patients affected by Ehlers-Danlos syndrome spondylodysplastic type 1 (formerly known as “EDS progeroid form”, MIM 130070), which is caused by galactosyltransferase I deficiency, an enzyme involved in the synthesis of the tetrasaccharide linker region, about 50% of decorin was secreted as a core protein in addition to its glycanated form [36,37]. Similarly, decorin underglycanation has been detected in the skin and bone of a mouse model of gerodermia osteodysplastica (MIM 231070) caused by defects in the *GORAB* gene encoding for a component of the COPI-coated vesicles responsible for retrograde transport from the Golgi apparatus to the ER and trafficking between cisternae within the Golgi apparatus [38,39]. Conversely, in a COG4 variant causing Saul–Wilson syndrome (MIM 618150), decorin from fibroblasts of affected individuals showed a greater proportion of higher molecular weight components compared with control cells [40]. In DBQD1 patients, as well as in our mutant mouse, no clinical phenotype has been observed or reported in the skin. For this reason, we analyzed the biochemical properties of skin decorin in mutant mice. Interestingly, we demonstrated that the decorin glycanation defect was not limited to cartilage, but also skin decorin showed a reduced hydrodynamic size, demonstrating that Cant1 was relevant for PG synthesis not only in cartilage where chondrocytes had huge sugar demands for GAG synthesis but also other tissues with modest Golgi glycosylation needs.

The main cartilage PG, aggrecan, was analyzed by immunoblotting as was performed for decorin. Because of its huge molecular weight due to a ~250 kDa core protein and more than one hundred GAG chains, only the core protein was analyzed. However, based on the decorin results, we expect that in aggrecan, the GAG chains in mutants are shorter compared with wild types. Moreover, we previously reported that the hydrodynamic size of chondroitin sulfate GAGs synthesized by chondrocytes after metabolic labeling with ^35^S-sulfate was reduced [14]. As observed for decorin, only glycanated aggrecan was secreted by chondrocytes in the medium since a positive signal for aggrecan was present only after digestion by chondroitinase ABC to remove the GAG chains and unmask the core protein epitope for the antibody. Indeed, in femoral head cartilage, glycanated aggrecan was present, as also confirmed by toluidine blue staining of epiphyseal sections, presenting no metachromatic differences in mutant compared with wild-type mice. Interestingly, in both *Cant1* knock-out and wild-type chondrocyte lysates, the unglycanated aggrecan form was observed, suggesting that once aggrecan has been glycanated, it is secreted. To further characterize the role of CANT1 in ECM protein synthesis, we also considered the collagen fraction. In both wild-type and mutant animals, collagen type II was normally expressed, and no collagen type I was detected, confirming the cartilage phenotype. Collagen post-translational modifications, including lysine and proline hydroxylation and hydroxylysine glycosylation, occur in the ER before the assembly of the collagen alpha chains in the collagen triple helix. The collagen triple-helix formation limits the post-translational modifications; in fact, in some variants of Osteogenesis Imperfecta, the collagen folding delay causes post-translational over-modifications, resulting in broader collagen bands by SDS-PAGE [41]. In the *Cant1* knock-out mouse, SDS-PAGE of collagen type II from cartilage was normal, suggesting that, within the limit of the technique, Cant1 did not affect collagen post-translational modifications. The same result was observed when collagen type I from the skin was analyzed.

Due to the interplay between aggrecan, decorin, and collagen type II, we studied the immunolocalization of the three ECM proteins in the tibia epiphysis at P4. We observed an altered distribution of aggrecan in the epiphysis of mutant compared with wild-type mice. In wild-type mice, a low aggrecan signal was detected in the region that gives rise to the secondary ossification center, whereas in mutants, a more homogeneous distribution of aggrecan throughout the whole epiphysis was observed. This likely reflects a delay in the formation of the secondary ossification center, as previously noted in mutant mice in the first 3 weeks of age [14]. Moreover, a higher decorin signal was observed between the chondrocyte columns of the proliferative zone in wild-type mice compared with mutant animals. Decorin is localized in the interterritorial matrix and is involved in ECM organization and integrity [42,43]. Its canonical function in collagen type I-rich tissues (tendon and skin) is to regulate fibril diameter and interfibrillar spacing; however, its structural role in cartilage has not been completely elucidated yet. Even if it has been demonstrated that decorin bound collagen type II either in vitro [44] or in cartilage biopsies [45], no changes in average fibril diameter have been observed in the decorin knock-out mouse [46]. Since decorin can bind to collagen fibrils and aggrecan core protein, it has been demonstrated that decorin plays a crucial role in maintaining the structural integrity of aggrecan rather than regulating collagen fibrillogenesis [46]. This finding might explain the even normal distribution of collagen type II observed in *Cant1* knock-out epiphysis by immunohistochemistry.

## 5. Conclusions

In this study, we used primary chondrocytes and cartilage from a mouse model of DBQD1 to obtain insight into the role of CANT1 in the synthesis of PGs and in the pathogenesis of DBQD1, which are mainly unknown. We demonstrated that the GAG synthesis defect due to CANT1 impairment does not result in the secretion within the ECM of PGs in the unglycanated form as has already been observed in other disorders of PG synthesis. However, the CANT1 defect leads to the synthesis of decorin with shorter GAG chains compared with wild-type animals. Finally, immunohistochemical studies in epiphyseal sections of mutant mice at P4 demonstrated that the PG structural defects mildly affect decorin, aggrecan, and collagen type II distribution in the ECM. Even if PGs play a crucial role in articular and endochondral cartilage, the structural alteration observed in the mutant mouse does not fully explain the skeletal defects observed in DBQD1. Due to the putative roles of CANT1 in Golgi glycosylation and protein quality control and to the multiple functions of the Golgi, further research is required to unravel the mechanisms underlying the DBQD1 phenotype and whether this enzyme might be harnessed to ameliorate the clinical phenotype.

## Data Availability

Data are available within this article or Supplementary Materials.

## Supplementary Materials

The following supporting information can be downloaded at https://www.mdpi.com/article/10.3390/biom14091064/s1. Figure S1: Toluidine blue staining of epiphyseal sections from *Cant1* knock-out and wild-type mice. Figure S2: Uncropped Western blots shown in Figures. Figure S3: Representative negative controls for immunohistochemistry studies.
