# Supplementary material for: Cant1 Affects Cartilage Proteoglycan Properties: Aggrecan and Decorin Characterization in a Mouse Model of Desbuquois Dysplasia Type 1
Source: Biomolecules. 2024 Aug 26;14(9):1064. doi: 10.3390/biom14091064 (PMC11430760; doi:10.3390/biom14091064)
Supplement: Supplementary file 1 [file biomolecules-14-01064-s001.zip › biomolecules-3097677-supplementary.pdf]

Figure S1

Cant1$^{+/+}$ *Cant1*$^{-/-}$

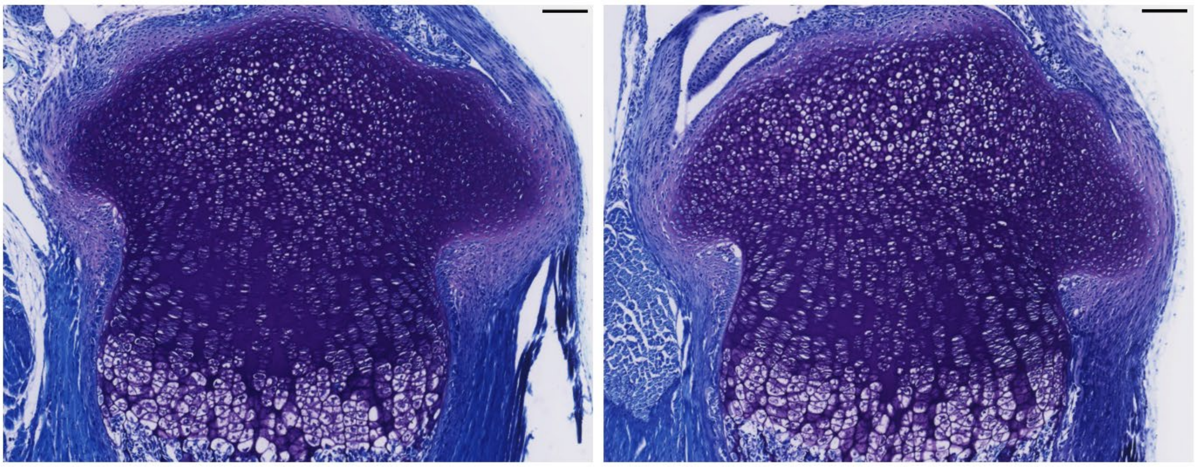

Figure S1. Toluidine blue staining of proximal tibia epiphysis of wild-type (*Cant1*$^{+/+}$) and mutant (*Cant1*$^{-/-}$) mice at P4. Histological study of epiphyseal cartilage was performed on formalin-fixed, paraffin-embedded 5 µm thick sections by toluidine blue staining as previously reported (Paganini et al, 2019). The cartilage matrix of *Cant1*$^{-/-}$ mice showed a normal intensity of toluidine blue staining compared with *Cant1*$^{+/+}$ according to previous results in epiphyseal cartilage of P7, P14 and P21 *Cant1*$^{-/-}$ mice (Paganini et al, 2019). This finding confirmed that proteoglycans were secreted in ECM in the glycanated form as demonstrated by Western blots of decorin and aggrecan. The images are representative of three independent experiments. Scale bar = 100µm.

Figure S2

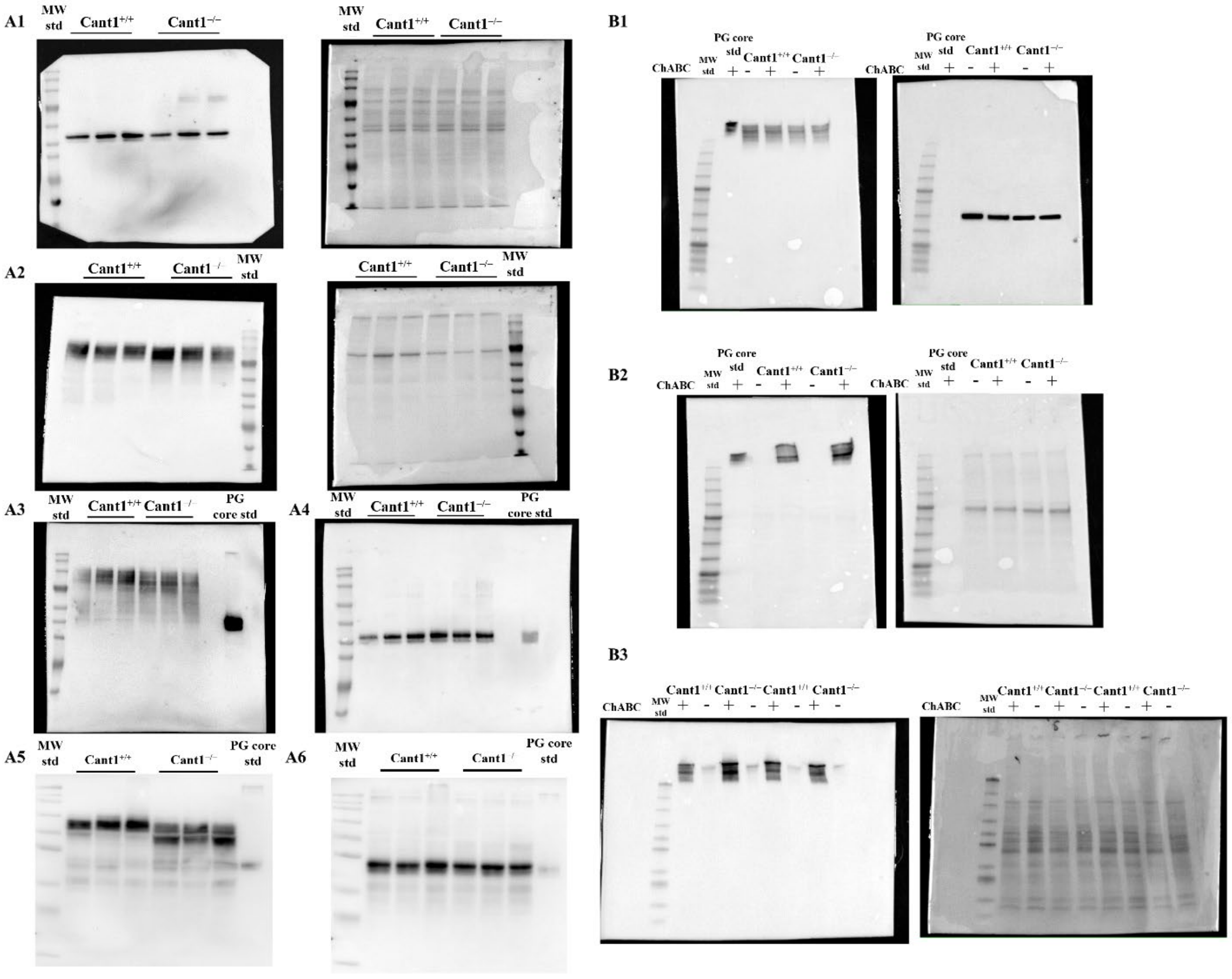

Figure S2. Original and uncropped images of decorin (A) and aggrecan Western blot (B).
(**A1**) Decorin western blot of cell lysates and (**A2**) culture medium from *Cant1* knock-out (Cant1$^{-/-}$) and wild-type (Cant1$^{+/+}$) chondrocytes. Membrane staining with Swift Membrane Stain™ was used as loading control. (**A3**) Decorin Western blot of femoral head cartilage extracts from *Cant1* knock-out (Cant1$^{-/-}$) and wild-type (Cant1$^{+/+}$) mice. (**A4**) Decorin Western blot of the same samples shown in (**A3**) after digestion with chondroitinase ABC to release the decorin core protein. (**A5**) Decorin Western blot of skin extracts from *Cant1* knock-out (Cant1$^{-/-}$) and wild-type (Cant1$^{+/+}$) mice. (**A6**) Decorin Western blot of the same samples shown in (**A5**) after digestion with chondroitinase ABC to release the decorin core protein. PG core std: standard of cartilage PG core proteins purified as described in Materials and Methods.
(**B1**) Aggrecan Western blot of chondrocyte lysates from *Cant1* knock-out (Cant1$^{-/-}$) and wild-type (Cant1$^{+/+}$) cells not digested (-) or digested (+) with chondroitinase ABC to release the core protein. β-actin was used as loading control. (**B2**) Aggrecan Western blot of chondrocyte culture medium from *Cant1* knock-out (Cant1$^{-/-}$) and wild-type (Cant1$^{+/+}$) cells not digested (-) or digested (+) with chondroitinase ABC to release the core protein. Membrane staining with Swift Membrane Stain™ was used as loading control. (**B3**) Aggrecan Western blot of femoral head cartilage extracts from *Cant1* knock-out (Cant1$^{-/-}$) and wild-type (Cant1$^{+/+}$) mice not digested (-) or digested (+) with chondroitinase ABC to release the core protein. Membrane staining with Swift Membrane Stain™ was used as loading control. PG core std: standard of cartilage PG core proteins purified as described in Materials and Methods; ChABC: chondroitinase ABC.

Figure S3

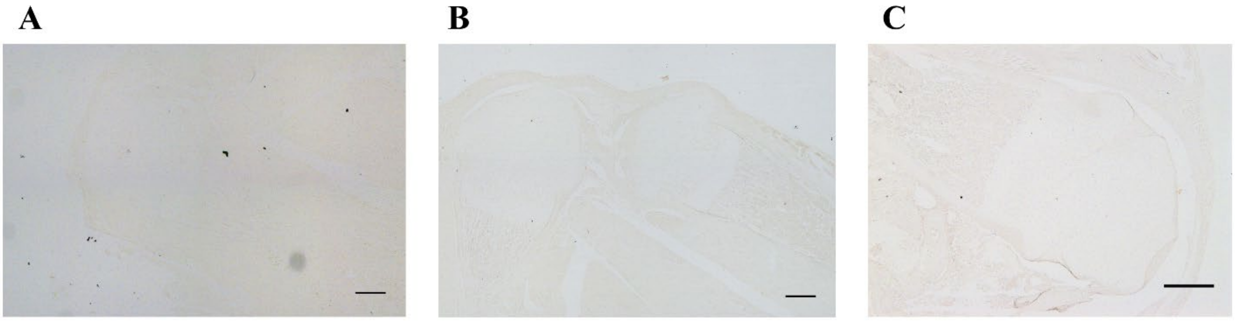

Figure S3. Representative negative controls of IHC studies
Immunohistochemistry of the proximal tibia epiphysis of P4 *Cant1* knock-out ($Cant1^{-/-}$) and wild-type ($Cant1^{+/+}$) mice. Negative controls were obtained following the standard IHC protocol but sections were not incubated with the primary antibodies to confirm the specific antibody signal in the other sections; sections immunostained for (**A**) decorin, (**B**) aggrecan and (**C**) collagen type II. Scale bar = 300 µm.

References

Paganini, C.; Monti, L.; Costantini, R.; Besio, R.; Lecci, S.; Biggiogera, M.; Tian, K.; Schwartz, J.M.; Huber, C.; Cormier-Daire, V.; et al. Calcium activated nucleotidase 1 (CANT1) is critical for glycosaminoglycan biosynthesis in cartilage and endochondral ossification. Matrix Biol. 2019, 81, 70–90. https://doi.org/10.1016/j.matbio.2018.11.002.
